# Draft Genome Sequences of 14 *Staphylococcus aureus* Sequence Type 5 Isolates from California, USA

**DOI:** 10.1128/genomeA.00098-17

**Published:** 2017-03-30

**Authors:** Samantha J. Hau, Darrell O. Bayles, David P. Alt, Tracy L. Nicholson

**Affiliations:** a Department of Veterinary Diagnostic and Production Animal Medicine, College of Veterinary Medicine, Iowa State University, Ames, Iowa, USA; b National Animal Disease Center, Agricultural Research Service, USDA, Ames, Iowa, USA

## Abstract

*Staphylococcus aureus* is part of the human epithelial microbiota; however, it is also a pathogen. The acquisition of mobile genetic elements plays a role in the virulence of *S. aureus* isolates and contributes to treatment failures. This report details the draft genome sequences of 14 clinical *S. aureus* isolates.

## GENOME ANNOUNCEMENT

*Staphylococcus aureus* is part of the microbiota found on the skin of humans and other animals. It is also a known pathogen that causes mild to severe infections, as well as toxin-mediated diseases (1). Methicillin-resistant *S. aureus* (MRSA) is a growing concern in the health industry, and MRSA isolates are categorized based on each isolate’s source as either hospital-acquired MRSA (HA-MRSA), community-acquired MRSA (CA-MRSA), or livestock-associated MRSA (LA-MRSA). Multilocus sequence typing is employed to determine an isolate’s sequence type (ST), which indicates the genetic background and denotes the characteristics of the isolate common to the group. The ST5 lineage is globally distributed and well known for acquisition of virulence factors and antibiotic resistance genes contained on mobile genetic elements (2).

We sequenced 14 ST5 isolates from patients with clinical disease caused by MRSA. These isolates were obtained from the University of California, Irvine (UCI3, UCI9, UCI11, UCI19, UCI21, UCI24, UCI27, UCI43, UCI45, UCI46, UCI48, UCI52, UCI56, and UCI64) (3). The patients had no known livestock exposure; however, because full patient histories were not provided, it could not be determined whether the isolates were HA- or CA-MRSA. Isolates were grown in Trypticase soy broth (BD Biosciences, Sparks, MD), and total genomic DNA was extracted using the High Pure PCR template preparation kit (Roche Applied Science, Indianapolis, IN).

Draft genome sequences were generated using an Illumina MiSeq instrument. Nextera XT DNA sample preparation and index kits (Illumina, San Diego, CA) were used to generate indexed libraries. Libraries were pooled and sequenced using the MiSeq version 2 500-cycle reagent kit, yielding 2 × 250-bp paired-end reads (Illumina).

Draft genome assemblies were generated using MIRA version 4.0.2 (http://mira-assembler.sourceforge.net/docs/DefinitiveGuideToMIRA.html), resulting in the average coverages indicated here for each isolate: UCI3, 56×; UCI9, 82×; UCI11, 72×; UCI19, 47×; UCI21, 66×; UCI24, 62×; UCI27, 40×; UCI43, 46×; UCI45, 53×; UCI46, 73×; UCI48, 46×; UCI52, 33×; UCI56, 36×; and UCI64, 52×. Following assembly, only contigs >1,500 bp in length having a coverage of >66% of the average genome coverage were retained. Additionally, when the assembly tool indicated that a contig was part of a potentially repetitive element, the contig was required to be >2,000 bp for inclusion in the assembly.

### Accession number(s).

The draft genome sequences obtained for these isolates were entered into DDBJ/ENA/GenBank with accession numbers as follows: UCI3, LKYU00000000; UCI9, LKZA00000000; UCI11, LKZC00000000; UCI19, LKZK00000000; UCI21, LKZM00000000; UCI24, LKZP00000000; UCI27, LKZS00000000; UCI43, LLAI00000000; UCI45, LLAK00000000; UCI46, LLAL00000000; UCI48, LLAN00000000; UCI52, LLAR00000000; UCI56, LLAV00000000; and UCI64, LLBD00000000.
